# 501. A Mobile Application for Longitudinal Social Participation Assessment and Resources Connection After Burn Injury

**DOI:** 10.1093/jbcr/irag033.160

**Published:** 2026-04-06

**Authors:** Huan Deng, Gregory Frasco, John T Morris, Amy L Acton, Diana L Tenney-Laperriere, Mary D Slavin, Lewis E Kazis, Colleen M Ryan, Jeffrey C Schneider

**Affiliations:** Spaulding Rehabilitation Hospital, Harvard Medical School; Boston University of Hariri Institute for Computing; Shepherd Center, Atlanta, GA; Phoenix Society for Burn Survivors; Boston University School of Public Health, Clarksville, TN; Massachusetts General Hospital Brigham – Spaulding; Boston University School of Public Health; Massachusetts General Hospital, Harvard Medical School; Spaulding Rehabilitation Hospital, Mass General Brigham, Harvard Medical School

## Abstract

**Introduction:**

Social participation is a critical but often challenging aspect of long-term recovery after burn injury. The Life Impact Burn Injury Recovery Evaluation (LIBRE) Profile is a well-established and psychometrically sound assessment to measure social participation in adult burn survivors. The traditional assessment method relies on infrequent clinic visits and paper-based questionnaires, which limit access and timely connection with resources. This study describes the development and pilot test of LIBRE GO!, a mobile application designed for longitudinal assessment of social participation and connection with resources in burn survivors.

**Methods:**

This was a clinical-academic-community collaboration, involving burn survivors, clinicians, technology experts, and researchers. The development process employed an iterative and user-centered approach. A pilot test using the developed LIBRE GO! was then conducted with a cohort of adult burn survivors (n = 8). Participants rated the Acceptability of Intervention Measure (AIM), Intervention Appropriateness Measure (IAM), and Feasibility of Intervention Measure (FIM), and attended focus groups to share their use experience.

**Results:**

LIBRE GO! mobile application was developed with four key functions: 1) LIBRE Profile assessment platform, 2) individualized score reports, 3) information about burn recovery, and 4) access to community services and resources. For the pilot test, average scores of the AIM, IAM, and FIM were 4.13, 4.28, and 4.31, respectively, where higher scores denoted greater satisfaction from 1 to 5. Participants reported that LIBRE GO! was easy to use as a valuable tool for tracking social engagement and connecting with resources. Optimizing the presentation of assessment scores and connection with individualized resources were suggested for future updates.

**Conclusions:**

LIBRE GO! is an accessible, feasible, and acceptable mobile application to longitudinally assess social participation and connect burn survivors with resources.

**Applicability of Research to Practice:**

Use of LIBRE GO! will identify individuals with social participation restrictions, empower survivors to manage their own recovery, and provide clinicians and researchers a tool to assess social participation outcomes and incorporate them into their care and investigations. Ultimately, these will advance the development of evidence-based approaches to improve social participation after burn injury.

**Funding for the study:**

This work was supported by the National Institute on Disability, Independent Living, and Rehabilitation Research (#90DPBU0008 and #90DPHF0004).

